# Bioluminescence Microscopy as a Method to Measure Single Cell Androgen Receptor Activity Heterogeneous Responses to Antiandrogens

**DOI:** 10.1038/srep33968

**Published:** 2016-09-28

**Authors:** Pallavi Jain, Bertrand Neveu, Lauriane Velot, Lily Wu, Yves Fradet, Frédéric Pouliot

**Affiliations:** 1 CHU de Québec - Université Laval Research Center, Quebec, Canada; 2 Department of Surgery (Urology), Faculty of Medecine, Laval University, Quebec, Canada; 3 Department of Molecular and Medical Pharmacology, David Geffen School of Medicine, University of California, Los Angeles, CA, USA; 4 Department of Urology, David Geffen School of Medicine, University of California, Los Angeles, CA, USA

## Abstract

Cancer cell heterogeneity is well-documented. Therefore, techniques to monitor single cell heterogeneous responses to treatment are needed. We developed a highly translational and quantitative bioluminescence microscopy method to measure single cell androgen receptor (AR) activity modulation by antiandrogens from fluid biopsies. We showed that this assay can detect heterogeneous cellular response to drug treatment and that the sum of single cell AR activity can mirror the response in the whole cell population. This method may thus be used to monitor heterogeneous dynamic treatment responses in cancer cells.

Metastatic cancer treatment options have made enormous advances in the area of targeted therapies. The number of efficient treatments now available are so many that it is driving the demand to customize therapy according to each patient’s cancer cell biology. In response to this growing demand for customized therapy, precise medical predicting tools have been designed to better assign tailored treatments for each patient. Currently, most predictive tools rely on analysis of biomarkers obtained from the patient’s bulk blood or tumor samples^1^,^2^. However, the known intratumoral cell heterogeneity in each patient may limit the capacity of whole tissue analysis to detect resistant or unresponsive cells^3^,^4^,^5^. For this reason, an assay to assess drug responsiveness in a single cell may be more accurate to determine patient response to targeted therapies.

In addition to tumor heterogeneity, another barrier to predict drug response is the number of possible resistance mechanisms used by cancer cells to escape anti-cancer-drug inhibitory effects^6^. Even if the sample is analyzed cell-by-cell, the interactions between many resistance genes is complex and cannot be completely predicted by static biomarkers based on genomic, proteomic, or transcriptomic parameters^7^,^8^,^9^.

One possible solution to circumvent these limitations would be to evaluate single cell drug sensitivity following drug exposure (dynamic assays). However, dynamic analysis is complicated when it involves the isolation and culture of primary cancer cells *ex vivo*. As an alternative to culture, we hypothesized that dynamic monitoring of a drug target modulation upon drug exposure in single cells could predict cell responsiveness and better differentiate resistant cells to drugs within a single output^10^,^11^. We further hypothesized that the integration of single cell response from fluid biopsies may better predict patient response to drugs.

Bioluminescence imaging has been largely exploited for gene promoter activity quantification and *in vivo* mice imaging^12^,^13^,^14^, but very few studies have taken advantage of bioluminescence microscopy to exploit it at the cellular level. Bioluminescence microscopy is a novel technique that uses the ability of reporter enzymes, named luciferases, to emit light with high energy after substrate addition. Because this enzymatic reaction needs ATP and substrate, only live cells expressing the reporter gene will produce light. Thus, the signal obtained is highly specific with no background^15^. All these parameters make bioluminescence microscopy a highly sensitive tool to accurately quantify promoter activity changes in single cells, but accuracy to monitor single cell promoter activity and drug response has not been characterized^15^,^16^,^17^.

To work towards a single cell dynamic assay to query prostate tumor cells directly, we developed and characterized a bioluminescence microscopy technique to measure androgen receptor (AR) activity in single cells upon antiandrogen treatment. Our overall findings showed that a single cell bioluminescence microscopy could indeed be performed to assess drug sensitivity with high accuracy, thus opening the door to the development of dynamic drug response assays in live circulating tumor cells from patients.

## Results

### Single cell bioluminescence microscopy imaging optimization after reporter system delivery

**Figure 1:**
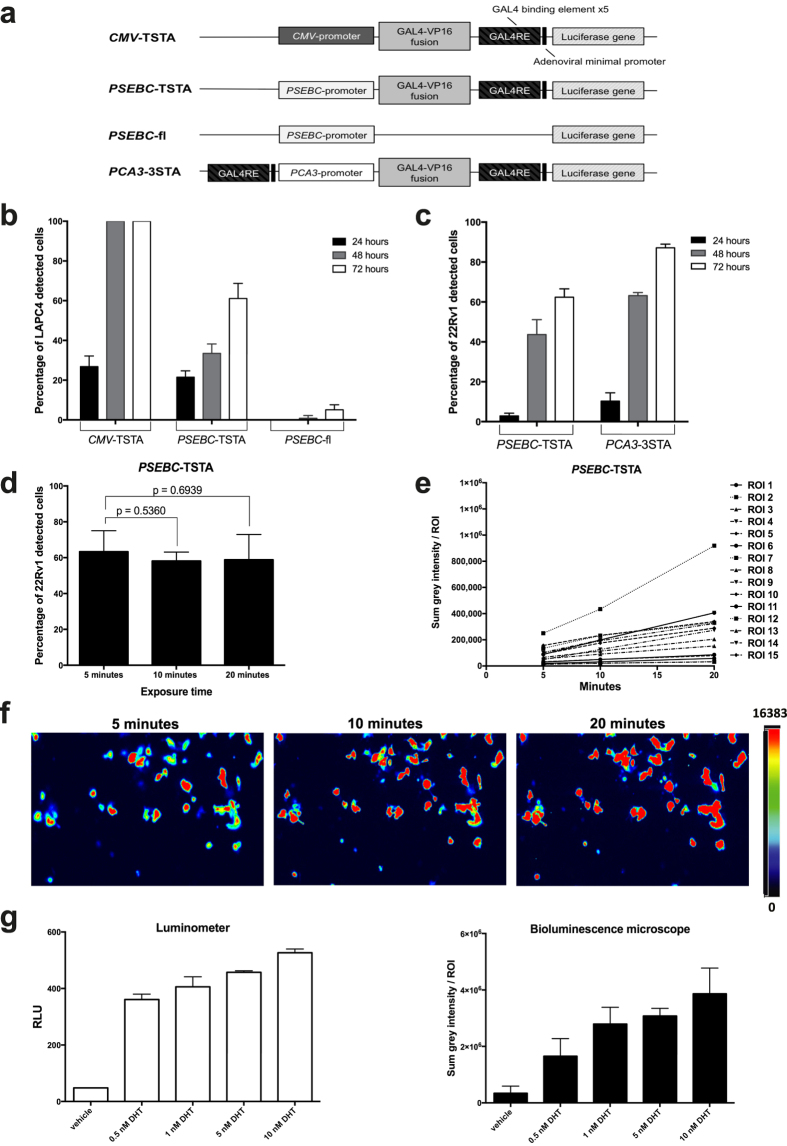
Optimization of a bioluminescence microscopy method for single cell imaging after adenoviral system transduction. **(a)** Scheme of the non-replicative adenoviruses used in the studies. **(b)** Amplification of the *PSEBC*-promoter signal by the Two-Step-Transcriptional Amplification system increases the number of AR-responsive PCa cells detected. LAPC4-GFP cells were transduced with 10^4^ infectious viral particles (ivp) of *CMV*-TSTA, *PSEBC*-TSTA or *PSEBC*-fl. Imaging was performed at 24, 48, and 72 h after D-luciferin addition and an exposure time of 5 min. **(c)** *PSEBC*-TSTA activity is more heterogeneous between single PCa cells when compared to a *PCA3*-promoter based imaging system (*PCA3*-3STA). 22Rv1-GFP cells were transduced using either *PCA3*-3STA or *PSEBC*-TSTA adenovirus and imaged after 72 h. The percentage of positive cells were analyzed as a ratio of luminescent over fluorescent cells. (**d)** Increasing the exposure time for imaging did not increase the number of cells detected after *PSEBC*-TSTA transduction. 22Rv1-GFP cells were transduced with *PSEBC*-TSTA. Seventy-two hours post-infection, imaging was performed at 20X magnification at exposure times of 5, 10, and 20 min. **(e)** Bioluminescence single cell microscopy is quantitative. Graph shows the linear increase in single cell (ROI 1-15) sum grey intensity over exposure time. **(f)** Representative images of 22Rv1 cells transduced with *PSEBC*-TSTA and plotted in (**e**) and showing that single cell luminescence increases with exposure time but not the number of detected cells. **(g)** Bioluminescence microscopy can titrate AR agonist DHT (0.5–10 nM) concentration ability to activate AR-transcription. LAPC4-GFP cells were infected with *PSEBC*-TSTA in media containing 0.5 to 10 nM of DHT. Seventy-two hours post-treatment, the cells were either lysed to be read by a conventional luminometer or imaged by bioluminescence microscopy (exposure time: 2 min). Sum grey intensity was normalized by number of fl-expressing cells (Sum grey intensity = sum grey intensity per ROI ÷ number of fl-positive cells). Firefly and GFP-expressing cells were counted using the cellSens software. Percentage of detected cells = (number of fl-positive cells ÷ number of GFP-expressing cells) × 100. Relative fl activity (RLU) was normalized by protein content (RLU = RLU/μg protein). Data represent technical triplicates ± SD.

With the goal of imaging primary prostate cancer (PCa) single cell response to antiandrogens, we first had to develop conditions for an appropriate imaging system driven by a promoter containing the androgen response elements sequence (ARE), which could be delivered into PCa cells. Because of high infectivity and thorough characterization in primary PCa cells, type 5 adenovirus was chosen as our delivery method^18^. For the PCa cell imaging using bioluminescence microscopy, we constructed type 5-adenovirus-enabling firefly luciferase (fl) expression driven by either a strong ubiquitous promoter (*CMV*), a well-characterized ARE-bearing promoter (*PSEBC*)^19^,^20^, or a PCa-specific androgen-insensitive promoter (*PCA3*) (Fig. 1a)^18^

As a first step to develop a quantitative bioluminescence imaging technique, we had to optimize the fl substrate (D-luciferin) concentration, infectious viral particles, and exposure time for signal intensity while minimizing toxicity. Using a transcriptional amplification system (TSTA) and a *CMV* promoter, we tested whether increasing D-luciferin concentration could enhance fl activity per region of interest (ROI). As shown in Supplementary Fig. 1a, optimal ROI sum grey intensity in 22Rv1 was achieved at a concentration of 3.5 mM of D-luciferin. When we increased the D-luciferin concentration up to 17.5 mM, the overall fl activity decreased by 30%, most likely secondary to cell toxicity (viability decreased to 40% with the highest dose (Supplementary Fig. 1a–c)). Because some dynamic bioluminescence studies would involve multi-well (many wells at the same time) and multi-condition (such as different exposure times) imaging, we also determined the signal sustainability over time following substrate exposure. When fl activity was quantified over time following *CMV*-TSTA transduction, we detected a sustained luciferase signal with no significant reduction from 20 min to 120 min following the addition of 3.5 mM of D-luciferin (Supplementary Fig. 1d). We also determined the optimal conditions for viral transduction and the amount of infectious viral particles (ivp) required to enable the detection of more than 90% of the cells, as the ultimate goal was to enable the detection of primary PCa cells in fluid biopsies (Fig. 1b and Supplementary Fig. 2). As shown in Fig. 1b and Supplementary Fig. 2, upon testing various amounts of infectious viral particles (10^3^–107 ivp), 10^4^ ivp of *CMV*-TSTA detected more than 90% of the cells in 3 out of the 4 PCa cell line populations tested, except DU-145 (AR−) PCa cells, thereby confirming this amount of adenovirus was appropriate for optimal transduction. However, in the DU-145 cell line, 90% of the cells were detected only with higher amounts (10^5^ ivp, Supplementary Fig. 2d). When the androgen-responsive *PSEBC*-fl adenovirus was tested at higher amount (10^7^ ivp), only 31, 10, and 57% of the cells were detected in 22Rv1, LNCaP, and LAPC4 cell lines, respectively, although cell viability was greatly affected at this amount (Supplementary Fig. 2a–c). Therefore, to increase fl reporter gene expression and detection rates, we cloned the *PSEBC* promoter in the TSTA system to generate the *PSEBC*-TSTA^19^ (Fig. 1a). *PSEBC*-TSTA detected more cells than *PSEBC*-fl virus did at 10^5^ ivp but much less than *CMV*-TSTA recorded. Only 73, 43, and 76% of the cells were detected in the 22Rv1, LNCaP, and LAPC4 cell lines, respectively (Supplementary Fig. 2a–c). As expected, no expression was observed in DU-145 (AR−) after 72 h of viral infection (Supplementary Fig. 2d). Interestingly, when we used the PCa-specific and androgen-insensitive *PCA3*-3STA^18^ promoter system for cell imaging, the percentage of detected PCa cells reached that of the *CMV*-TSTA (Fig. 1c). To further exclude confounding factors explaining heterogeneous single cell *PSEBC* activity within AR + cell lines, we analyzed whether exposure time could impact the number of detected cells. Fig. 1d–f and Supplementary Fig. 3 show that prolonging exposure time by 4-fold did not enhance the percentage of detected cells using either the *CMV*-TSTA (Supplementary Fig. 3a) or *PSEBC*-TSTA (Fig. 1d) system. However, increasing the exposure time did increase the sum of activity of each ROI (Fig. 1e and Supplementary Fig. 3b). Together, these results show that transduction (Fig. 1b,c), exposure time (Fig. 1d,f), or fl level of expression (Fig. 1b) could not explain absence of *PSEBC* activity in around 40% of the cells, depicting single cell heterogeneous activity in the same androgen-sensitive (AR+) PCa cell lines. We thus showed that the *PSEBC* promoter was inactive in many cells of AR sensitive PCa cell lines, even though the androgen sensitivity of these cell lines as a whole remained the same (Supplementary Figs. 4a–c). This showed that *PSEBC*-TSTA had the ability to specifically detect androgen-sensitive cells and there is a hidden androgen-insensitive population within the same AR + PCa cell line.

### Bioluminescense microscopy is highly quantitative

To measure single cell AR activity modulation by antiandrogens, we had to ensure that the bioluminescence microscopy deployed was indeed quantitative. By imaging single cells with increasing exposure times, we obtained a linear increase in the grey intensity with a mean activity ratio of 1.79- and 3.20-fold between 5 to 10 min and 5 to 20 min, respectively (Fig. 1e–f), depicting the high accuracy of bioluminescence microscopy following adenoviral transduction (y = 13961x − 7680, r^2^ = 0.9763). Enzalutamide (Enz) is a novel highly potent second generation-AR antagonist indicated for castration-resistant PCa, while bicalutamide (Bic) is a weaker, classical AR antagonist used in the early-stages of the disease. To further evaluate how our method could quantify AR activity modulation by androgens and antiandrogens, we compared it to the current gold standard used for bioluminescence quantification, namely luciferase assays on whole cell lysates using a luminometer apparatus^21^. We compared luminometer and bioluminescence microscopy quantifications in LAPC4 cells infected with *PSEBC*-TSTA and exposed to vehicle (ethanol), DHT, or DHT + Enz (Supplementary Fig. 5). As expected, following luminometer quantification, the normalized RLU was induced by DHT, an effect that was completely inhibited by enzalutamide (8.4 ± 0.65 and 0.72 ± 0.17-fold, respectively). Similarly, luminescence microscopy measured AR induction by DHT or complete inhibition by enzalutamide (7.80 ± 0.85 and 0.52 ± 0.18-fold, respectively). Because DHT is a direct agonist of AR, we also measured how bioluminescence microscopy could titer DHT concentrations (Fig. 1g). Again, bioluminescence measurements were linear over increasing doses of DHT (y = 0.044x + 1.03, r^2^ = 0.9512) (Fig. 1g, right panel). This linear coefficient was similar to that obtained with the luminometer (y = 0.11x + 1.26, r^2^ = 0.77) (Fig. 1g, left panel). Correlation between luciferase signal and DHT concentration were compared between bioluminescence microscope and luminometer using the Fisher’s Z-transformation. This comparison showed no significant difference (Fisher’s Z value = 0.6844, p 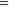 0.4937). Overall results indicate that bioluminescence microscopy was as quantitative as luciferase assays but provided the advantage of enabling single cell activity measures.

### Bioluminescence microscopy is able to quantify single cell heterogeneous response to antiandrogens

**Figure 2:**
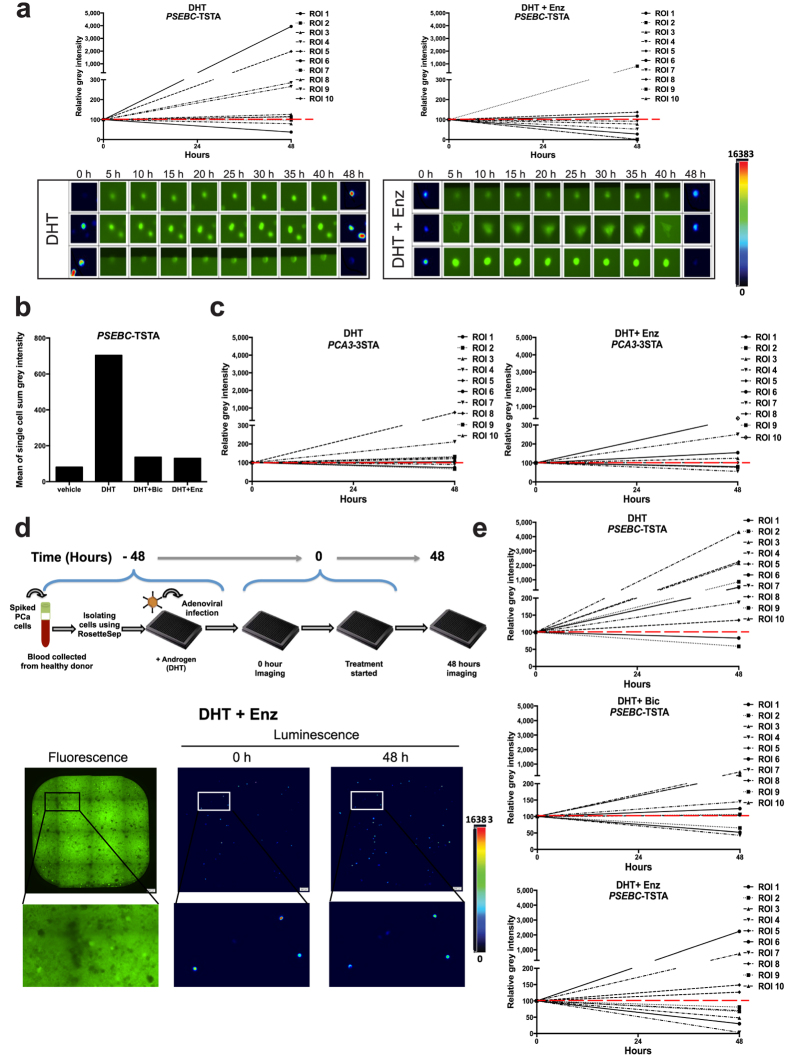
Imaging single cell heterogeneous responses to AR agonist and antagonists by using bioluminescence microscopy. **(a)** *PSEBC*-TSTA detected single cell heterogeneous responses to DHT and Enz in AR-responsive LAPC4 cells. LAPC4-GFP cells were infected with *PSEBC*-TSTA in media containing DHT (1 nM). Forty-eight hours post-infection, the cells were imaged after D-luciferin addition. After imaging, the media was changed and the treatments started (DHT (1 nM) or DHT + Enz (1 nM + 10 μM)). GFP biofluorescence imaging was then performed every 5 h to track the cells. After 48 hours of treatment, luciferase imaging was repeated to determine the change in fl expression. Lower panels show representative single cell luminescence signals before and after treatments. The corresponding cells tracked and imaged by biofluorescence microscopy is also shown. **(b)** Sum of single cell LAPC4-GFP activity upon AR agonist (DHT) or antagonist (Enz or Bic) treatment. **(c)** *PCA3* promoter activity is not modulated by antiandrogen treatment. LAPC4-GFP cells were infected with *PCA3-*3STA, treated and imaged as described in (a). **(d)** *Upper panel:* Scheme of the method used to isolate and image spiked PCa cells from blood. Spiked LAPC4-GFP cells were isolated from blood of a healthy donor and were infected with *PSEBC*-TSTA in media containing DHT (1 nM). Forty-eight hours post-infection, the cells were imaged and the media was changed to start the treatments (DHT (1 nM), DHT + Bic (1 nM + 10 μM) or DHT + Enz (1 nM + 10 μM)). Every 5 h, GFP biofluorescence imaging was performed to track the cells. At 48 h, luminescence imaging was repeated to determine single cell fl expression changes. *Lower panels:* Biofluorescence and bioluminescence images of blood spiked-LAPC4-GFP cells transduced with *PSEBC*-TSTA after PCa cell isolation. **(e)** Bioluminescence microscopy quantification of single cell responses to AR-antagonists after *PSEBC*-TSTA transduction of PCa spiked cells (LAPC4-GFP). Relative grey intensity = ((sum grey intensity per ROI − sum grey intensity of background) ÷ sum grey intensity at 0 h) × 100. Data represent technical triplicates ± SD.

To exploit the unique ability of the microscope to quantify cell-by-cell luminescence using an androgen-modulated *PSEBC* promoter, LAPC4 (AR+) PCa cells were cultured in DHT containing media with or without antiandrogens. Quantification of the single cell sum grey intensity revealed heterogeneous response patterns to DHT or antiandrogens within the same cell line (Fig. 2a, more data for LAPC4 and other cells in Supplementary Fig. 6). When LAPC4 cells were treated with AR agonist DHT, most of the cells (80%, n = 10) displayed an increase in fl activity, while the remaining 20% showed a decrease. In contrast, upon withdrawal of the androgens or under antiandrogen (Enz) treatment, most of the cells showed a decrease in fl activity (70%), with 30% showing an increase (Fig. 2a). Because single cell imaging relies on isolated cells, we wanted to ensure that the selected cells continued to maintain cell line androgen and antiandrogen responses and that the cells were not, for example, cells with aberrant responses. For each treatment group (Vehicle, DHT, DHT + Bic, DHT + Enz), we calculated the sum of fl activity of ten single cells analyzed following *PSEBC*-TSTA transduction and bioluminescence microscopy quantification (single cell sum of activity in Fig. 2a). As shown in Fig. 2b, similar to the LAPC4 cell line, the single cells (also LAPC4) were strongly induced by DHT (8.8-fold), an induction inhibited by both Bic and Enz. These results show that single cells sampled and analyzed were representative of the overall cell line hormonal responsiveness and that luminometer luciferase assays represented the sum of a heterogeneous cell population, with some being inhibited and some being induced. To ensure that the transcriptional responses observed following the hormonal treatments of DHT, Enz or Bic were not due to their indirect effect of transcriptional inhibition on cell viability, we tested the method on AR + 22Rv1 cells. These cells have a functional AR pathway but are resistant to antiandrogen growth inhibition but may still be modulated by Enz and Bic (Supplementary Fig. 4a–c)^22^. Supplementary Fig. 6c shows that 22Rv1 single cell fl activity was still modulated by both the androgens and antiandrogens, demonstrating that the short-term treatment effect was secondary to the AR transcriptional modulation by AR ligands such as Enz. As an additional control, we tested hormonal treatments on cells transduced with *PCA3*-3STA, an androgen-insensitive system (Fig. 2c). We observed that *PCA3* promoter-dependent single cell bioluminescence activity did not show induction nor inhibition on addition of DHT or DHT + Enz. The overall fold change with *PCA3* promoter-dependent activity for 10 cells on addition of DHT or DHT + Enz was 1.75- and 1.09- fold, respectively (Fig. 2c), showing again that the treatment effects observed earlier using *PSEBC*-TSTA were secondary to the transcriptional modulation by AR ligands. Furthermore, to translate single cell bioluminescence microscopy quantification methods into a clinical application, we used *PSEBC*-TSTA to target spiked cancer cells isolated from the blood of healthy individuals. In this way, we ensured that this optimized method could image single cells harvested from blood in a heterogeneous cell population. Following the enrichment of blood with cancer cells, cells were transduced with *PSEBC*-TSTA and cultured for 48 h in the presence of Enz or Bic. As shown in Fig. 2d, the spiked cells were targeted with the adenovirus (*PSEBC*-TSTA), which enabled the detection of 60–77% of the cells (luminescent cells over fluorescent cells). In addition, when isolated cells were treated with Bic or Enz, despite the presence of remaining blood cells, we were able to image the antiandrogen AR transcriptional response with high specificity, as all of the bioluminescent cells (LAPC4 or LNCaP) were also fluorescent (Fig. 2d,e and Supplementary Fig. 7). Noticeably, LNCaP cells (which are highly sensitive to Enz) showed a homogeneous response to Enz (Supplementary Fig. 7), while the response of isolated LAPC4 cells was highly variable (Fig. 2e).

## Discussion

In this study, we describe a novel method to measure single cell drug sensitivity using bioluminescence microscopy to link single live-cell heterogeneous responses to therapy to whole cell population sensitivity. As proof of concept, we show that bioluminescence microscopy can quantitatively titrate androgen and antiandrogen effects on androgen receptor activity following the transduction of an androgen-responsive promoter driving a transcriptional amplification system and a luciferase reporter. Through dynamic single cell imaging, we also show single cell response to AR agonist or antagonists to be highly heterogeneous in the same cell line. For instance, up to 30% of the cells showed an increase in luciferase signal when exposed to AR antagonists (e.g. enzalutamide) in androgen-sensitive cell lines. Furthermore, the sum of single cell activities correlates with overall cell line activity as shown in Fig. 2a,b, demonstrating that this deconstructive method can successfully identify differentially responding populations hiding within an androgen-responsive cell population. Finally, we confirm by studying single cell response in spiked cancer cells, that the potential of the technique can be further expanded to target cancer cells from fluid biopsies and can be developed as a treatment response-predicting tool for personalized medicine.

With the changing outlook toward cancer management, it has been shown that the progression of the diseased state leads to clonal selection and cellular heterogeneity due to mutations, stochastic variations, or environmental protuberances^23^. This is secondarily reflected at the genomic, transcriptomic, or proteomic level. All the above stated factors, in turn, lead to cancer treatment failure and disease recurrence, as the treatment targeting one cancer cell population may prove to be ineffective against another^5^,^24^. Most currently used single cell analysis and biological tools are based on fixed cell staining and cytological methods^25^. These involve static DNA or RNA-based analysis for whole genome characterization which provides an enormous amount of information on the genetics of individual cells^8^,^9^,^26^,^27^. The amount of data generated from these studies is significant and difficult to analyze because each single cell phenotype is unknown; hence there is a growing need to study the response phenotypes of single cells^28^,^29^. We thus put forth that dynamic transcriptional bioluminescence imaging will help to link responsive phenotypes to large data omics analysis, which when studied further, can enable the discovery of new alterations or the integration of a panel of alterations found only in unresponsive single cells.

The method presented herein has the unique advantage of being highly integrative at the molecular, cellular, and whole cell population levels. We show that bioluminescence microscopy analysis is highly quantitative and can therefore detect molecular-level changes, integrate many antiandrogen resistance mechanisms (resistome), and reveal the absence of AR inhibition by such drugs, cell-by-cell. Because this assay is dynamic upon antiandrogen exposure, it takes into account all AR antagonist resistome actors and their interactions to escape from antiandrogen inhibitory effects. Indeed, antiandrogen resistome gene mutation, amplification, and expression or epigenetic changes are integrated as a bioluminescent signal that is measurable for each cell and can be further validated using fixed cell analysis. This constitutes an advantage over single cell omics analysis which can only study alterations in one particular cellular component (transcriptomics, genomics, epigenomics, proteomics) due to methodological limitations^30^. At cellular levels, our method can image heterogeneous responses to a drug and integrate it thereafter to determine cell population sensitivity. Because most techniques to analyze fluid biopsies described thus far are neither quantitative nor dynamic, transcriptional imaging by bioluminescence microscopy is unique, as it enables for quantitative baseline drug target activity (e.g. AR active or inactive) and its dynamic modulation by a drug (antiandrogens). As shown in Fig. 2, the single cell analysis of antiandrogen-sensitive cell line LAPC4 reveals a cell sensitivity gradient rather than clusters of sensitive and insensitive cells. This highlights the importance of obtaining quantitative single cell assays to determine individual sensitivity within a group of cells. Because we expect circulating tumor cells to be highly heterogeneous, with a variable drug response, the capability of this method to integrate heterogeneous cell responses into an overall response is highly translational. It may thus complement advances made in circulating tumor cell isolation and characterization by enabling a linkage between clonal resistance and clinical response to therapy. In addition, with transcriptional bioluminescence dynamic microscopy, single cell sensitivity is defined by drug target (AR) modulation due to a drug (antiandrogens) and sensitivity determination does not rely on cell growth, the latter being a strong technical barrier for dynamic drug sensitivity testing when using primary cancer cells. Moreover, this direct drug target assay requires shorter culture times and does not incorporate its cell growth surrogate and associated non-specific gene expression changes.

This study did have certain limitations. With the developed system, we were able to detect only AR-expressing cells. Therefore, the cell population that did not express AR was not detected which could contribute to cell line resistance. We also did not characterize the cell-by-cell resistance phenotypes of cells experiencing an increase in activity upon antiandrogen treatment, as a cell in which an ARE is not modulated by an antiandrogen is unlikely to be sensitive to such a drug.

In summary, our results show that bioluminescence transcriptional single cell microscopy allows not only for dynamic, integrative, and quantitative drug response measurements, but also a better visualization of single cell responses and ultimately, heterogeneous drug response within a tumor cell population. If applied to single cells from fluid biopsies, this method may be useful to predict treatment responses and create a link between single cell treatment response and single cell omics analysis.

## Material and Methods

### Plasmid construction and adenoviral production

Adenoviral plasmids for *PSEBC*-TSTA, *PCA3*-3STA, and *CMV*-TSTA were constructed as previously described^18^. *PSEBC*-fl was devised using gateway cloning for adenoviral constructs. The *PSEBC* promoter was PCR-amplified from pENTR-L1R5-*PSEBC*-GAL4VP16 and inserted into a pENTR-L1R2 backbone plasmid to build *PSEBC*–fl. Lentivirus-expressing renilla luciferase as well as GFP were constructed using the plasmid pccl-*CMV*-RL-IRES-EGFP^12^. Five μg of pccl-*CMV*-RL-IRES-EGFP plasmid and three helper plasmids (Gag-Pol, Rev and VSV-G) were transfected into 293T cells using lipofectamine 2000 (Life Technologies, Burlington, ON, Canada), in a 60 mm Petri dish. Virus particles were collected thereafter and titrated using serial dilutions and GFP-positive cells were counted by means of fluorescent activated cell sorting (FACS).

### Cell cultures

22Rv1 and LNCaP (prostate cancer cell lines) were cultured in RPMI-1640 media containing 10% fetal bovine serum (FBS). LAPC4 (prostate cancer cell line) and HEK 293 (human embryonic kidney cells) were cultured in DMEM media containing 10% FBS. DU-145 (prostate cancer cell line) was cultured in EMEM media containing 10% FBS. LAPC4 and DU-145 were kindly provided by Dr C. Sawyers and Dr L. Old respectively. Other cell lines were obtained from ATCC. The cell lines were tested for absence of mycoplasma using the MycoAlert Mycoplasma Detection kit (Lonza, Basel, Switzerland).

### Production of stable transduced cell lines

22Rv1, LAPC4, LNCaP, and DU-145 were seeded in a 24-well plate (10,000 cells/well). Twenty-four hours later, the lentivirus was transduced at a multiplicity of infection (MOI) of 5 along with polybrene (8 μg/ml). Twenty-four hours post-infection, the media was changed to remove the virus and the cells were kept in culture until we obtained more than 60% of GFP expressing cells. The cells were then trypsinized and collected in PBS containing 2% FBS. GFP-positive cells were sorted by means of FACS to obtain 22Rv1-GFP, LAPC4-GFP, LNCaP-GFP and DU-145-GFP. These cells were maintained in culture for one passage to propagate before using them in experiments.

### Bioluminescence microscopy

Bioluminescence imaging was performed using an Olympus LV200 microscope equipped for luminescence imaging, transmitted brightfield and transmitted fluorescence imaging. Samples to be imaged were seeded in a 384-well black plate (Ibidi, Madison, WI, USA) and placed on a motorized stage (Prior Scientific, Rockland, MA, USA) provided with a stage-top incubator (Tokai Hit, Fujinomiya, Japan). Luminescence imaging was performed using either 20X (5 min of exposure per field of view (FOV)) or 40X (15 s of exposure per FOV) objectives. The emitted photons passed through an open channel without filter and collected onto an electron-multiplying CCD camera (Andor Ixon 897). For the biofluorescence imaging, samples were excited at a wavelength of 470 nm (X-Cite XLED1, Excelitas Technologies, City, MA, USA) and the fluorescence emission of eGFP was collected using the CCD camera. Data analysis and process design for automated image capture were achieved using the cellSens software (Olympus, Tokyo, Japan).

### Adenoviral infection and viability assay

22Rv1, LAPC4, LNCaP, and DU-145 cells (1,000 cells/well) were seeded in a 384-well black plate. Twenty-four hours later, the cells were transduced with either *CMV-*TSTA, *PSEBC*-TSTA*, PSEBC*-fl or *PCA3*-3STA adenovirus. Imaging was performed with 3.5 mM of D-luciferin (re-suspended in PBS) (Caliper Lifesciences, Hopkinton, MA, USA). Media was refreshed at each time point (24, 48, and 72 h) and imaging was done with an exposure of 5 min per FOV by means of the LV200 bioluminescence microscope. The percentage of detected cells was defined as the number of bioluminescent over biofluorescent cells (GFP-positive cells) multiplied by 100. At the end of the protocol, following imaging, 5 μl of PrestoBlue reagent (ThermoFisher Scientific, Waltham, ON, Canada) was added to each well in 45 μl of media and incubated overnight at 37 °C in a cell culture chamber. The media was then collected and fluorescence was measured by Fluoroskan Ascent (ThermoFisher Scientific) at the excitation/emission wavelength of 540/595 nm. Cell viability was defined as follows: (fluorescence of infected cells − fluorescence media) ÷ (fluorescence control non-infected cells - fluorescence media) × 100.

### D-luciferin concentration optimization

22Rv1 cells were seeded in a 384-well black plate and 10^4^ infectious viral particles (ivp) of *CMV*-TSTA adenovirus were added 24 h later. Seventy-two hours after infection, 0.88, 1.75, 3.5, 8.75, and 17.5 mM of D-luciferin were added into separate wells and the sum grey intensity was recorded thereafter every 5 min for 2.5 h using the time lapse registering protocol of the cellSens software. Sum grey intensity was first quantified for bioluminescence-positive cells at each concentration in a defined ROI and then normalized by the number of luciferase-positive cells. The viability assay was performed at 72 h only, following the imaging.

### Exposure time optimization

22Rv1 cells were seeded in a 384-well black plate and either *CMV*-TSTA or *PSEBC-*TSTA adenovirus was added 24 h later. Seventy-two hours after infection, D-luciferin (3.5 mM) was added to each well and after 20 min, imaging was performed with exposures of 5, 10 and 20 min in a defined frame. The percentage of detected cells was calculated as described above. Sum grey intensity was quantified using the cellSens software in the same ROI at different exposure times.

### Androgen responsiveness assessment by luciferase assay

22Rv1, LAPC4, and LNCaP cells were seeded in 24-well plates. Twenty-four hours later, 10^4^ ivp *PSEBC*-TSTA adenovirus was diluted in 50 μl of media containing 10% charcoal-stripped FBS (FBS-CT) and treated with either vehicle (Ethanol), dihydrotestosterone (DHT) (0.5–10 nM, as indicated), DHT + Bic (1 nM + 10 μM) (Sigma-Aldrich, St. Louis, MO, USA), or DHT + Enz (1 nM + 10 μM) (MedChem Express, South Brunswick, NJ, USA). Forty-eight hours after treatment, bioluminescence microscopy or luciferase assays were used to measure luciferase activity. For the luciferase assays, the cells were lysed using a passive lysis buffer (Promega, Madison, WI, USA) and luciferase activity was measured by means of Luminoskan Ascent (ThermoFisher Scientific) following the addition of D-luciferin, as stated in the Dual-luciferase protocol (Promega). Relative fl activity (RLU) was normalized by protein content in each well (RLU = RLU ÷ μg of protein). Protein content was estimated by adding 250 μl of Bradford reagent (ThermoFisher Scientific) to 3 μl of total lysate. Absorbance was then read using an Infinite F50 absorbance microplate reader (TECAN, Mannedorf, Switzerland) at 595 nm. For the bioluminescence microscopy, 3.5 mM of D-luciferin in fresh media was added to each well and imaging was performed with an exposure time of 2 min per FOV. Sum grey intensity was normalized by the total number of counted fl-expressing cells (Sum grey intensity = sum grey intensity per ROI ÷ number of fl-positive cells). Sum grey intensity was calculated using the cellSens software.

### Single cell treatment response

22Rv1, LAPC4, LNCaP, and DU-145 cells were seeded in a 384-well black plate. Twenty-four hours later, the cells were transduced with 10^4^ infectious viral particles of *PSEBC*-TSTA or *PCA3*-3STA in media containing 1 nM of DHT and 10% FBS-CT. Forty-eight hours after infection, the cells were imaged by bioluminescence microscopy and after the treatment was added (vehicle, DHT (1 nM), DHT + Bic (1 nM + 10 μM) or DHT + Enz (1 nM + 10 μM)). To track the change in position and growth of single cells in which fl activity was measured, biofluorescence microscopy imaging was performed every 5 h for 48 h (GFP-positive cells) after which time bioluminescence imaging and quantification were repeated on the same cells. Sum grey intensity was then determined for single cells before and after treatment using the cellSens software. Relative grey intensity = ((sum grey intensity per ROI − sum grey intensity of background) ÷ sum grey intensity at 0 h) × 100.

### Single cell treatment response from spiked cancer cells isolated from blood

Blood was collected from a healthy donor and placed in a heparin-coated tube. This study was approved by the Institutional Review Board of the CHU de Quebec Hospital, Quebec, QC, Canada. Informed consent was obtained by the donor for blood sampling. All experiments were performed in accordance with relevant guidelines and regulations. LAPC4 and LNCaP cells were added to the blood sample (500 cells/3 ml of blood), followed by a custom-made RosetteSep cocktail (STEMCELL Technologies, Vancouver, BC, Canada) at a volume of 50 μl per ml. The sample was gently mixed and incubated thereafter at room temperature for 20 min. Fifteen milliliters of Ficoll-Paque Plus (GE healthcare Life Sciences, Mississauga, ON, Canada) were then placed in a 50 ml SepMate tube (STEMCELL Technologies)^31^ and blood was poured gently along the walls of the tube onto the Ficoll layer. The tubes were subsequently centrifuged at 1200 *g* for 20 min at room temperature with the brake on. The top 10 ml of the top layer was removed and the remaining top layer was gently transferred to clean 50 ml tubes. Forty milliliters of PBS containing 2% FBS were then added and the tubes were centrifuged at 350 *g* for 8 min with the brake on. The supernatant was then gently removed, leaving behind 1 ml in each tube. The pellets were then resuspended in 5 ml of RPMI-1640 media containing 10% FBS-CT and DHT(1 nM) and the tubes were centrifuged at 350 *g* for 8 min with the brake on. The resulting supernatant was then gently removed to reduce the final volume to 50 μl. The recovered cells were then seeded in a 384-well plate. Cells were transduced with 5 × 10^4^ viral particles of PSEBC-TSTA. The plate was kept in a shaker overnight. Forty-eight hours after infection, bioluminescence imaging was performed and after the treatments were added (vehicle, DHT (1 nM), DHT + Bic (1 nM + 10 μM) or DHT + Enz (1 nM + 10 μM)). The cells were tracked every 5 h for 48 h using biofluorescence microscopy. Forty-eight hours later, bioluminescence imaging was repeated on the same cells. Percentage of targeted cells = (number of bioluminescence positive cells ÷ number of GFP positive cells) × 100. The sum of grey intensity values was determined for single cells before and after treatment using cellSens software. Relative grey intensity = ((sum grey intensity per ROI − sum grey intensity of background) ÷ sum grey intensity at 0 h) × 100.

### Statistical analysis

All of the statistical analyses were conducted using the two-sided t-test with Welch’s correction, with (*) indicating p ≤ 0.05. The variance was consistant within each experimental groups. The Fisher’s Z test was used to compare the correlations.

## Additional Information

**How to cite this article**: Jain, P. *et al*. Bioluminescence Microscopy as a Method to Measure Single Cell Androgen Receptor Activity Heterogeneous Responses to Antiandrogens. *Sci. Rep.* **6**, 33968; doi: 10.1038/srep33968 (2016).
