# Corrigendum: Bioluminescence Microscopy as a Method to Measure Single Cell Androgen Receptor Activity Heterogeneous Responses to Antiandrogens

**DOI:** 10.1038/srep37381

**Published:** 2016-11-21

**Authors:** Pallavi Jain, Bertrand Neveu, Lauriane Velot, Lily Wu, Yves Fradet, Frédéric Pouliot

Scientific Reports 6: Article number: 33968; 10.1038/srep33968 published online: 09 28 2016; updated: 11 21 2016.

The Acknowledgments section of this Article is incomplete:

“We thank N. Bisson (Department of Cell and Molecular Biology, Laval University) for revision of the manuscript. This project was supported by Movember Canada and Prostate Cancer Canada (PCC Movember New investigator pilot grant 2012-933 and Rising Star Grants RS2014-04), the Canadian Urological Association Scholarship Funds, the Fonds de recherche du Québec – Santé (FRQS) for clinician-scientists (FRQS-22830), and the John R. Evans Leaders Fund from Canadian Foundation for Innovation (32441). L.W. is supported by the CDMRP Prostate Cancer Research Program award W81XWH-15-1-0256”.

should read:

“We thank N. Bisson (Department of Cell and Molecular Biology, Laval University) for revision of the manuscript. This project was supported by Movember Canada and Prostate Cancer Canada (PCC Movember New investigator pilot grant 2012-933 and Rising Star Grants RS2014-04), the Canadian Urological Association Scholarship Funds, the Fonds de recherche du Québec – Santé (FRQS) for clinician-scientists (FRQS-22830), and the John R. Evans Leaders Fund from Canadian Foundation for Innovation (32441). F.P. has received a grant from Astellas Canada for the development of a real-time dynamic bioluminescent microscopy platform. L.W. is supported by the CDMRP Prostate Cancer Research Program award W81XWH-15-1-0256”.

